# A Rare Pulmonary Complication of Esophageal Carcinoma: A Case of an Acquired Esophago-Pulmonary Fistula

**DOI:** 10.7759/cureus.89665

**Published:** 2025-08-09

**Authors:** S Jesica, M Logeshwari, Rajoo Ramachandran, Vignesh Kumar

**Affiliations:** 1 Radiology, Sri Ramachandra Institute of Higher Education and Research, Chennai, IND

**Keywords:** esophageal cancer, esophago-pulmonary fistula, lung abscess, sems, thoracic imaging

## Abstract

Esophageal-respiratory fistulae are abnormal communications between the esophagus and the respiratory tract, most commonly appearing as tracheoesophageal or bronchoesophageal fistulas. Esophago-pulmonary fistulas represent a rare subtype, typically associated with malignancy, and may lead to severe complications such as lung abscesses. We report a case of a 58-year-old male patient who presented with a two-week history of fever, foul-smelling mucoid sputum, dyspnea, dysphagia, and weight loss. Imaging revealed a distal esophageal mass with a fistulous communication into the right lower lobe and a lung abscess. Bronchoscopy confirmed purulent secretions, and upper gastrointestinal endoscopy showed a malignant esophageal rent. Biopsy diagnosed squamous cell carcinoma. The patient was started on empirical antibiotics, antifungals, and total parenteral nutrition. Positron emission tomography computed tomography (PET-CT) demonstrated a distal esophageal tumor with a possible skip lesion and a direct fistula into the abscess cavity. A covered self-expandable metallic stent (SEMS) was inserted for palliation. On follow-up, tumor ingrowth above the stent with a persistent leak required consideration of secondary stenting. The patient was deemed unfit for radiotherapy and was referred for chemotherapy. This case highlights an uncommon but life-threatening complication of esophageal carcinoma. Prompt diagnosis through imaging and endoscopy is essential to guide appropriate palliative interventions. In inoperable cases, stenting combined with supportive care remains the mainstay of treatment. Recognizing this rare presentation is important for improving outcomes in patients presenting with overlapping gastrointestinal and pulmonary symptoms.

## Introduction

Esophageal-respiratory fistulae (ERFs) are abnormal connections between the esophagus and the respiratory system. While tracheoesophageal and bronchoesophageal fistulas are more common, esophago-pulmonary fistulas are rare [1-3]. Malignancies of the esophagus, lung, or mediastinum are the most frequent causes of acquired ERFs [4]. Patients often present with recurrent aspiration pneumonia, malnutrition, and respiratory distress, leading to high morbidity and mortality. This has importance in cancers involving the mid-third of the esophagus, where the primary mass is in close proximity to the trachea and bronchus [5]. Early diagnosis is critical due to the potential for rapid clinical deterioration. Imaging modalities such as chest CT, endoscopy, and bronchoscopy are essential for detection. Here, we present a case of distal esophageal squamous cell carcinoma with direct invasion into the right lower lobe, forming an esophago-pulmonary fistula and resulting in a lung abscess.

## Case presentation

A 58-year-old male patient presented with a two-week history of cough producing foul-smelling whitish mucoid sputum, fever with chills, shortness of breath, dysphagia, regurgitation, and weight loss. Baseline investigations were unremarkable.

Bronchoscopy revealed purulent secretions in the right lower lobe. Bronchial wash was sent for acid-fast bacilli smear, GeneXpert, bacterial and fungal cultures, cytology, and galactomannan testing. Empirical antibiotics (piperacillin-tazobactam, metronidazole) and nebulized bronchodilators were initiated. The bronchoalveolar lavage (BAL) fungal smear showed budding yeast cells; galactomannan optical density (OD) index was 1.3 (normal cut-off value <0.5), prompting the initiation of voriconazole (6 mg/kg IV 12th hourly loading followed by 4 mg/kg IV 12th hourly maintenance). BAL fungal culture was negative.

**Figure 1:**
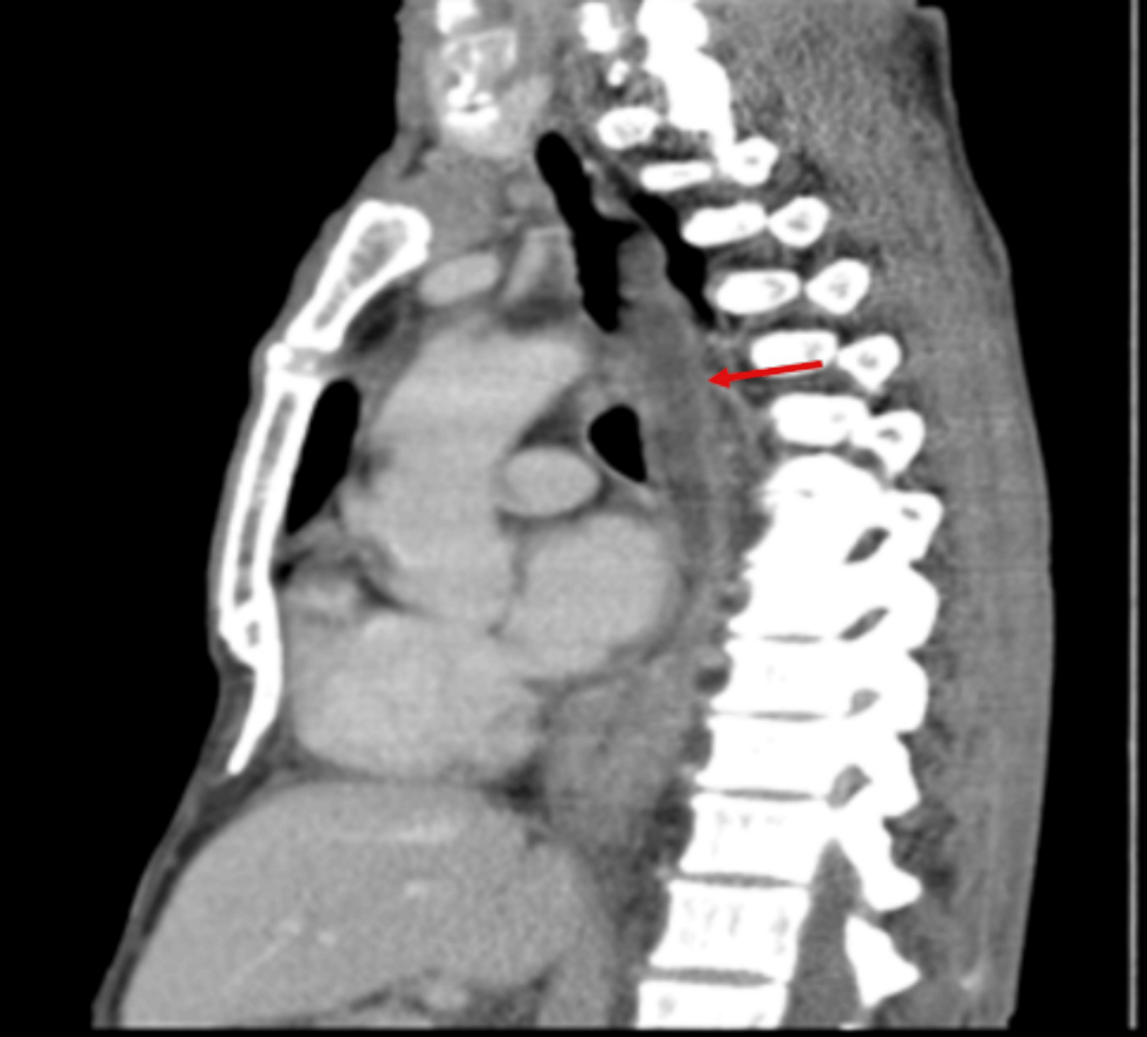
CT venous phase - sagittal section The red arrow shows the circumferential enhancing wall thickening of the distal esophagus.

**Figure 2:**
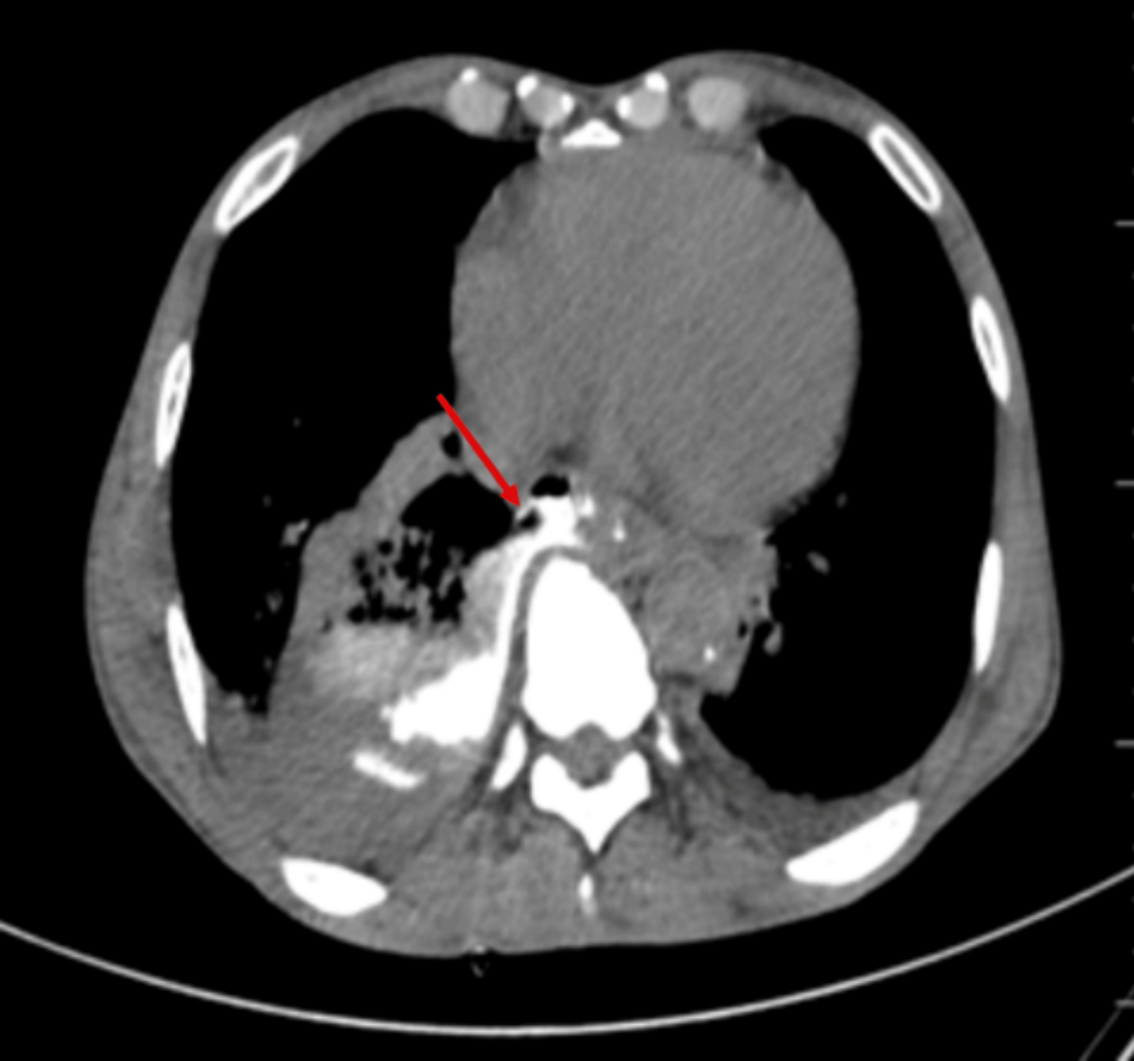
CT oral contrast phase - axial section The red arrow indicates the contrast tracking forming a fistulous communication into the lung parenchyma, resulting in the formation of a round, relatively well-defined, thick-walled peripheral enhancing collection in the right lower lobe parenchyma.

**Figure 3:**
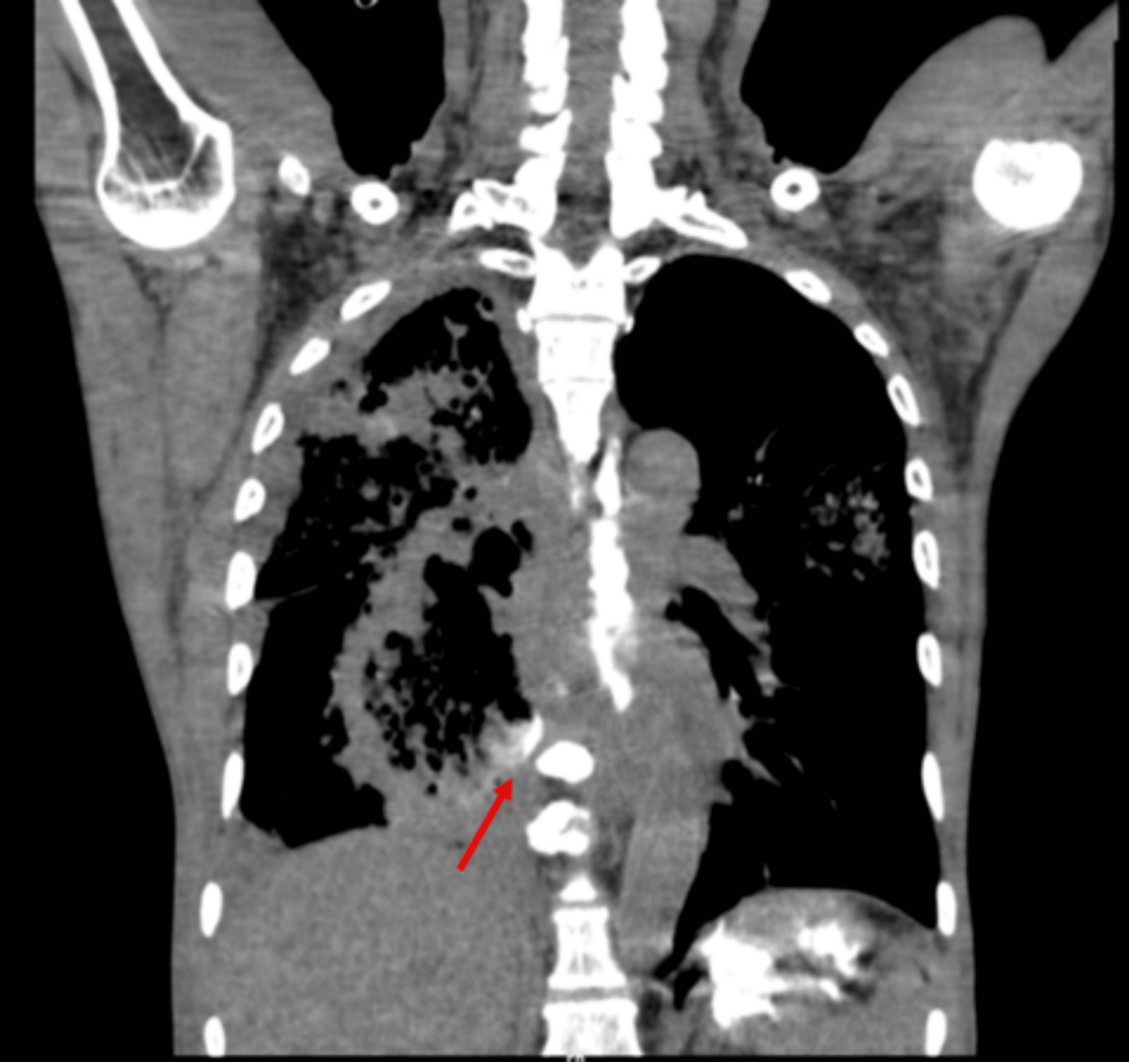
CT oral contrast phase - coronal section The red arrow shows contrast leak into the right lung parenchyma.

Contrast-enhanced CT (CECT) of the thorax revealed a distal esophageal mass with an esophago-pulmonary fistula and right lower lobe lung abscess. Bilateral upper lobe reticular opacities and multifocal consolidation were also noted. Oral contrast confirmed the fistula (Figures 1-3).

**Figure 4:**
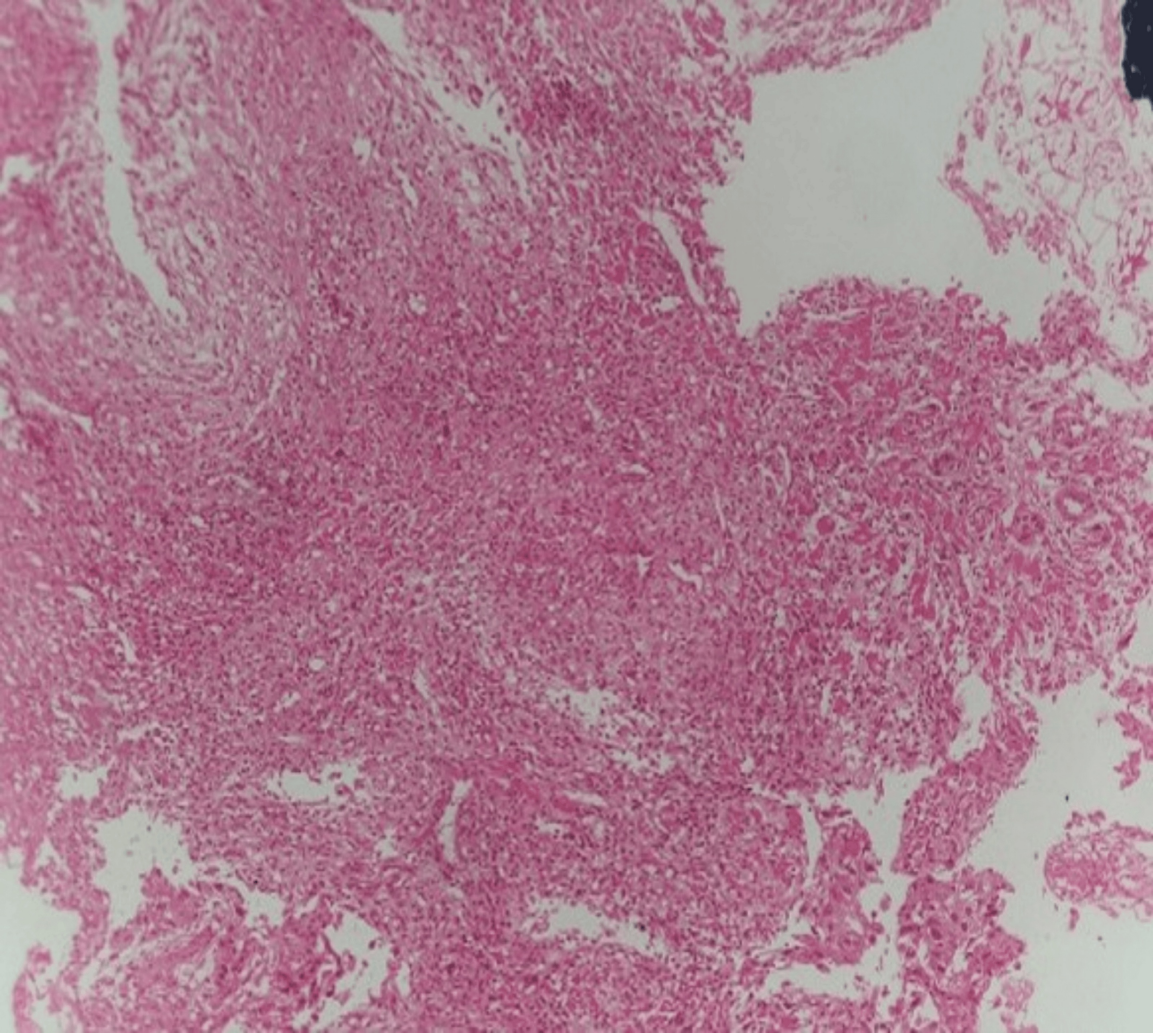
Histopathology image using hematoxylin and eosin stain (H&E), magnification 100x The histopathology image shows infiltration of tissue fragments by moderately differentiated squamous cell carcinoma with adjacent fragments of necrosis.

Upper gastro-intestinal endoscopy showed a malignant esophageal growth with a rent; biopsy confirmed squamous cell carcinoma (Figure 4). The patient was kept nil per oral and started on total parenteral nutrition (TPN).

Positron emission tomography computed tomography (PET-CT) demonstrated circumferential thickening of the distal thoracic esophagus with a fistulous communication to a right lower lobe lung abscess, and a possible skip lesion in the cervical esophagus. There were bilateral pleural effusions, fibrotic changes in both lungs (right > left), and mediastinal and hilar lymphadenopathy.

**Figure 5:**
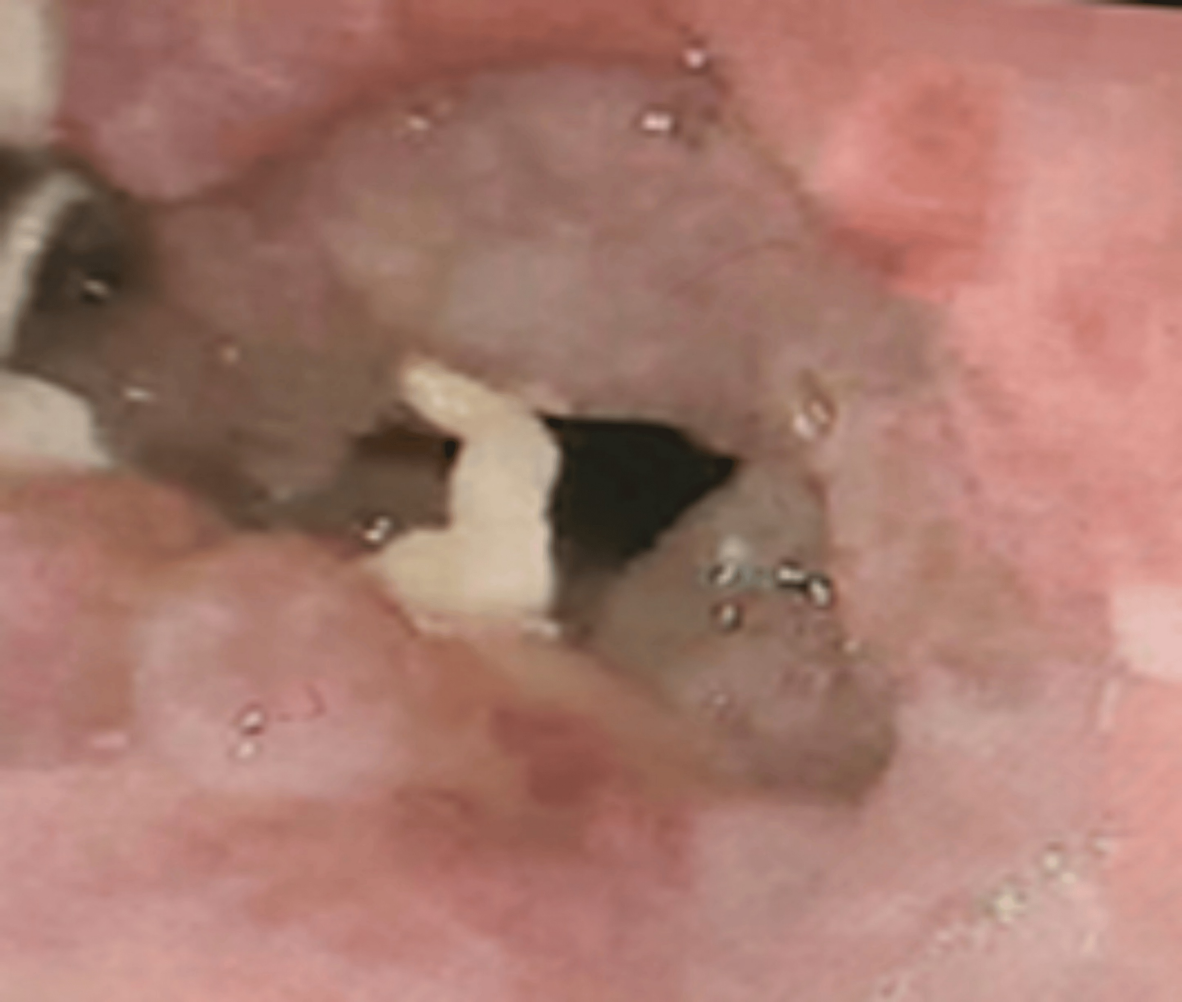
Endoscopy image showing the esophageal stent

**Figure 6:**
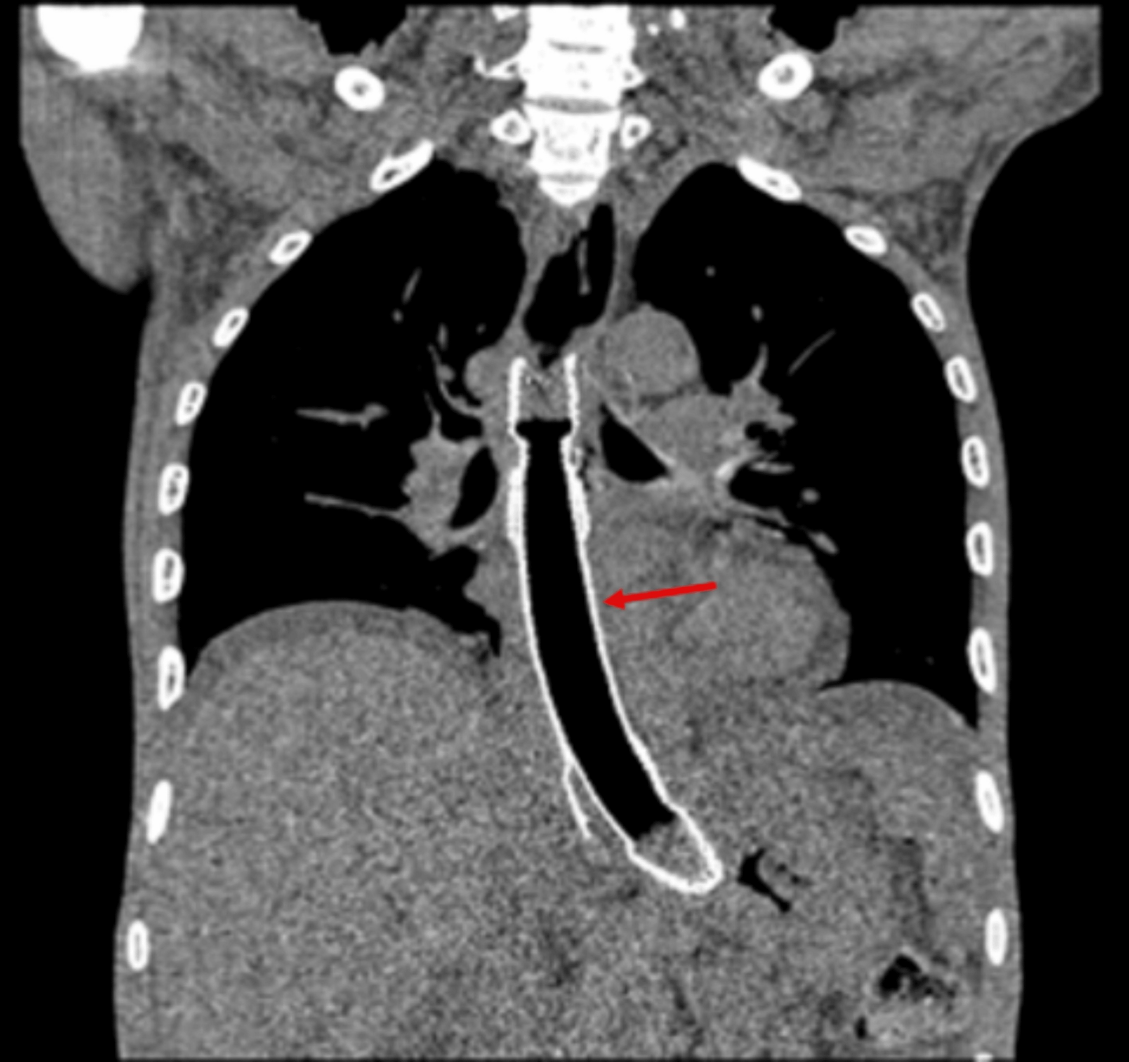
Plain CT - coronal section The red mark shows the esophageal stent in situ at the level of D4-D11 vertebral bodies.

Following multidisciplinary evaluation, a covered self-expandable metallic stent (SEMS) was placed for palliation (Figures 5-6). The patient continued on TPN post-stenting.

**Figure 7:**
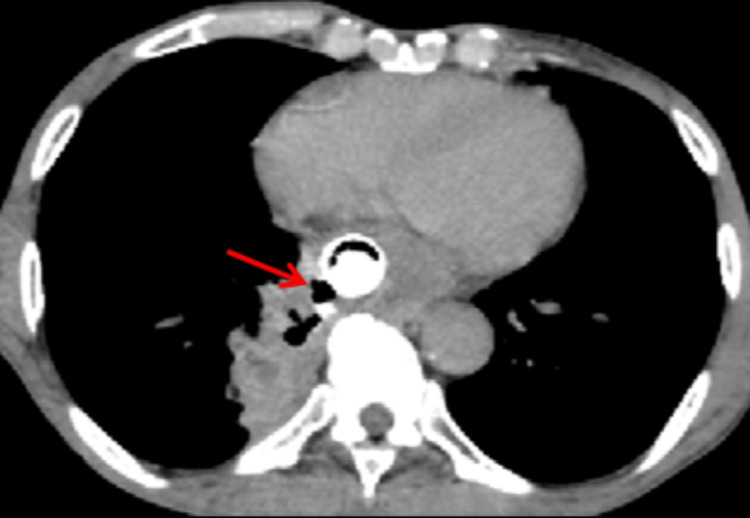
CT contrast phase - axial section showing contrast leak and luminal wall thickening

On follow-up admission, radiation oncology deemed the patient unfit for radiotherapy. Surgical oncology recommended decortication after tumor board review. Repeat CECT thorax with oral contrast showed an in-situ distal esophageal stent with two areas of extraluminal contrast, suggesting stent leakage (Figure 7). There was intraluminal wall thickening at the proximal end of the stent.

Upper gastro-intestinal endoscopy showed tumor ingrowth above the SEMS, causing mild luminal narrowing. A second stent (stent-in-stent) was advised on follow-up. Medical oncology recommended chemotherapy with capecitabine.

## Discussion

This case illustrates a rare complication of esophageal squamous cell carcinoma, an esophago-pulmonary fistula leading to lung abscess. Acquired ERFs are often due to malignancy, caustic ingestion, prolonged intubation, granulomatous infections, or prior esophageal surgery, and also reported in the use of antiangiogenic drugs such as bevacizumab [4,6]. TEFs with pulmonary fistulas comprise 3-11% of all cases and are predominantly located in the middle third of the esophagus, while distal fistulas, such as in this case, are less common [2,7].** **Occasionally, some patients might present with symptoms of pneumonitis due to lung abscess by esophageal pulmonary fistula and later found out to be esophageal carcinoma [8].

ERFs require urgent management due to risks of aspiration pneumonia, respiratory failure, and sepsis. Imaging plays a pivotal role in diagnosis and management planning. CT with oral contrast helps confirm fistulae, while multi-detector CT and 3D images provide accurate characterization of ERFs in adults in a wide variety of clinical scenarios, with certain findings helpful in surgical planning [9]. It is particularly valuable in detecting ERFs, as it allows simultaneous assessment of both the esophageal lumen and surrounding structures. Compared to other imaging modalities, it provides better anatomical detail, helps localize the fistula tract, detect associated abscesses, and evaluate the extent of disease, thereby guiding therapeutic planning [4].

Flexible esophagoscopy and bronchoscopy are needed to confirm the diagnosis and anatomic assessment of the suspected fistula that aids in providing additional information for treatment planning [10]. Video fluoroscopy and thin-slice CT with water-soluble contrast aid in subtle or equivocal cases, minimizing complications such as pulmonary edema or mediastinitis associated with barium aspiration.

Management goals include sealing the fistula, treating infection, and restoring nutrition [11,12]. Non-surgical management is preferred in malignant ERFs. Options include SEMS placement, jejunal or parenteral feeding, and targeted antimicrobial therapy. Surgery is reserved for select cases with adequate fitness.

SEMS is the most common palliative approach. Potential complications include stent migration, tumor ingrowth, leakage, pain, bleeding, and reflux. Secondary stenting (stent-in-stent) may be required in cases of tumor overgrowth or persistent leakage, as observed in this patient.

## Conclusions

Acquired esophageal-respiratory fistulas in adults, though rare, are serious and life-threatening complications of esophageal malignancy. A high index of suspicion is warranted in patients presenting with dysphagia, weight loss, and recurrent pulmonary infections. Early diagnosis through imaging and endoscopy is critical to initiate timely treatment. In inoperable cases, palliation with endoscopically placed covered stents and nutritional support remains the mainstay of management. A multidisciplinary approach involving gastroenterology, thoracic surgery, pulmonology, oncology, and radiology is essential to optimize care. Given the high morbidity and limited available evidence, further prospective studies are needed to establish standardized guidelines for effective management.
